# The Effect of Telehomecare on Patients’ Health-Related Quality of Life, Satisfaction, Disease Self-Management Skills, Provider Satisfaction, and Informal Caregiver Strain: Longitudinal Cohort and Cross-Sectional Study

**DOI:** 10.2196/70809

**Published:** 2026-01-15

**Authors:** Troy Francis, Aleksandra Stanimirovic, Sonia Meerai, Nida Shahid, Valeria E Rac

**Affiliations:** 1 Program for Health System and Technology Evaluation Toronto General Hospital Research Institute University Health Network Toronto, ON Canada; 2 Institute of Health Policy, Management and Evaluation University of Toronto Toronto, ON Canada; 3 School of Social Work Laurentian University Sudbury, ON Canada

**Keywords:** chronic obstructive pulmonary disease, heart failure, longitudinal data analysis, patient-reported outcomes, remote patient monitoring, telehomecare

## Abstract

**Background:**

Heart failure (HF) and chronic obstructive pulmonary disease (COPD) are responsible for a significant amount of the economic and chronic disease burden that impacts the Ontario health system. Telehomecare, a home self-management program launched by the Ontario Telemedicine Network (OTN), was created to improve access to quality care and limit health care use. However, few data are available on patient-, caregiver-, and provider-reported outcomes of telehomecare.

**Objective:**

This study aims to evaluate the impact of the OTN telehomecare program on the health-related quality of life (HRQoL), disease-management skills, and satisfaction of patients with HF and those with COPD; informal caregiver strain index; and nurse satisfaction with telehomecare.

**Methods:**

We used a prospective longitudinal cohort design, including patients with HF and those with COPD enrolled in Ontario’s telehomecare program, informal caregivers of patients in the program, and nurses providing services in telehomecare. Patients and informal caregivers were administered telephone surveys at baseline, month 3, month 6, and month 12 follow-up from July 2016 to December 2019. The outcomes for the longitudinal surveys were patient-perceived HRQoL, disease self-management skills, perception of telehomecare (ease of use and usefulness), satisfaction with telehomecare, and informal caregiver-perceived strain. Cross-sectional surveys were conducted with nurses to assess nurse perception and satisfaction with telehomecare. Participant data were analyzed using general linear mixed models in SAS Statistical Software (version 9.4; SAS Institute Inc).

**Results:**

Overall, a total of 194 patients (HF, n=117; COPD, n=77), 62 caregivers, and 24 nurses participated, with an overall response rate of 51% (280/551). The average age of patients with HF and those with COPD was 71 (SD 11.3) years and 70 (SD 11.1) years, respectively, and 52% (100/194) were men. A significant improvement in overall HRQoL was observed among patients with HF at month 12 (−18.37, *P*<.001). Minimal clinically important differences were observed across all HRQoL domains for people with HF, indicating clinically meaningful improvement over the study period. No statistically significant improvement in HRQoL was observed among patients with COPD; however, minimal clinically important differences were observed in the physical functioning dimension. Patients reported being confident in self-managing their diseases throughout the study, but as patients aged, their perception of and satisfaction with telehomecare was shown to decrease (*P*=.002 and *P*=.002, respectively). Caregivers reported relatively low strain scores (mean 10.3, SD 5.9) throughout the program, and nurses reported moderate levels of satisfaction (mean 6.7, SD 1.5) with telehomecare at follow-up.

**Conclusions:**

In this population, telehomecare demonstrated an ability to improve the HRQoL of patients with HF and those with COPD. However, the long-term sustainability of HRQoL improvements in patients following telehomecare requires further investigation. Furthermore, telehomecare was shown to decrease informal caregiver-perceived strain, and nurses described moderate levels of satisfaction and perceived quality of care with telehomecare.

## Introduction

Chronic diseases are the leading cause of death and disability in Ontario and require continued comprehensive care and monitoring. Heart failure (HF) and chronic obstructive pulmonary disease (COPD) are responsible for a significant amount of the economic and chronic disease burden that impacts the Canadian health system [1-3]. An estimated 1 million Canadians are currently living with HF [4], which is projected to have a direct cost of CAD $2.8 billion (US $2.03 billion) per annum, with hospital readmissions due to HF costing nearly CAD $500 million (US $365 million) a year [5]. COPD is estimated to be the sixth leading cause of mortality in Canada, with the national burden estimated to be CAD $1.5 billion (US $1.09 billion), and exacerbations projected to cost between CAD $650 million (US $474.5 million) and CAD $740 million (US $540.2 million) annually for moderate and severe events, respectively [6]. Patients with HF and those with COPD have been known to have poor health-related quality of life (HRQoL), diminished life expectancy, suffer from frequent acute exacerbations, and experience various other comorbidities, such as other cardiovascular diseases, diabetes, cancer, depression, osteoporosis, and malnutrition [7,8]. Informal caregivers play a central role in the health outcomes of patients with HF and those with COPD, but caregiving has also been shown to have a substantial impact on caregiver outcomes. Informal caregivers of patients with HF and those with COPD face complex situations; besides providing practical help, caregivers face physical, psychological, and social burdens in providing care and support [9,10]. Additionally, caregiving has been associated with an increased risk of depression, mortality, and poor quality of life [10,11].

Telemedicine and virtual care are innovative solutions to chronic disease management [12,13]. The use of remote patient monitoring allows for targeted follow-up and improved continuity of care [14]. Telemedicine programs can improve patient self-management and increase self-efficacy [15], quality of life, and satisfaction [16]. Telemedicine programs have been successfully implemented in various countries around the world [17]; however, before the COVID-19 pandemic, Canada’s adoption of telemedicine lagged behind most other nations [18]. The pandemic has accelerated the global adoption of telemedicine, changing how people in Canada access health care by speeding up digital transformation. Many patient consultations, both primary and specialist, are now conducted virtually [19].

The Ontario telehomecare program was launched in 2007 through collaboration with the Ontario Telemedicine Network (OTN), Ontario Ministry of Health and Long-Term Care, and the Canada Health Infoway to improve access to quality care for patients with HF and those with COPD and limit potential health care use [20]. Telehomecare, a 6-month home self-management program delivered by nurses using health coaching and remote patient monitoring, allows patients with chronic conditions the opportunity to be actively involved in their care [20,21]. The results of the program’s clinical outcomes, implementation, and adoption have been reported elsewhere [3,22-24]. However, the long-term perceptions of the patients’ quality of life and self-management, as well as those of caregivers and providers involved within telehomecare, have not yet been reported, highlighting the need for further longitudinal research. The importance of highlighting patient-centered care in designing and implementing a comprehensive telehomecare program allows for greater attention to detecting sudden changes in overall health, which patients find most important. Patient-reported outcomes, such as HRQoL, are reports coming directly from patients about how they feel concerning a health condition and its therapy, without interpretation of their responses by a clinician or caregiver [25]. Patient reported-outcomes are important because they provide patients’ perspectives on treatment benefits and provide another opportunity to measure treatment benefits beyond survival, disease, and physiological markers [26].

This study aimed to (1) evaluate the impact of the OTN telehomecare program on HRQoL, disease self-management skills, and satisfaction with the program among patients with HF and those with COPD and (2) determine informal caregiver strain and nurse satisfaction with telehomecare.

This study highlights the importance of not only focusing on patient outcomes but also on informal caregivers and telehomecare nurses when evaluating an intervention. Understanding the OTN telehomecare’s impact requires a comprehensive view of all stakeholders involved. Informal caregivers assist with transmitting the clinical data daily to the telehomecare nurses, who monitor the data and ensure patient safety. Examining these stakeholders helps to understand how the technology influences health outcomes and support systems, leading to more effective, patient-centered care.

## Methods

### Ethical Considerations

Before engaging potential study participants, the intervention evaluation protocol was approved by the Research Ethics Board of the University Health Network (Reference No: 16-5136). Ethics approval was secured from 77 sites, including hospitals, Community Care Access Centers, community health centers, and family health teams participating in the telehomecare program across 8 local health integration networks (LHINs). The evaluation study team contacted potential patient participants by telephone, and if they agreed to participate, verbal consent was obtained using a telephone consent script approved by the Research Ethics Board. All other stakeholders who agreed to take part in the study provided consent either in person or by telephone. Participants who provided consent received a copy of the study information and consent form and were informed that the research findings would be published in reports, articles, and presentations. All study data were anonymized and deidentified.

### Study Design and Population

This study followed the Strengthening the Reporting of Observational Studies in Epidemiology (STROBE) guidelines [27]. This study used a prospective longitudinal cohort and cross-sectional design, and the population included patients with HF or COPD enrolled in the telehomecare program, telehomecare nurses, and informal caregivers participating in telehomecare across 8 LHINs in Ontario from July 2016 to December 2019. The LHINs were created to plan, coordinate, integrate, and fund health services at the local level and are separated into different geographical catchment areas. Patients and caregivers were referred to the program through the LHINs or self-referrals and were screened for program eligibility criteria by telehomecare staff. Patients and caregivers were approached and purposively selected to ensure variation in sampling from each LHIN (urban vs rural) and disease type. Those patients and caregivers who agreed to participate were administered longitudinal surveys. The OTN, in collaboration with the LHIN engagement leads, referred telehomecare nurses to the evaluation study team. Nurses who were delivering telehomecare at the time of the evaluation were considered and approached to participate in the cross-sectional surveys.

### Eligibility for Telehomecare

Patients and caregivers qualified with a documented HF or COPD diagnosis, with or without comorbidities. “Heavy users” had recent respiratory or cardiac hospitalizations or multiple emergency visits. They also may have been receiving nursing services or had frequent primary care visits. Eligibility included being aged 18 years and older, capable of consent, fluent in English, able to operate telehomecare equipment, and residing in a private or retirement home with a landline. Additional criteria for the intervention study required consent to telehomecare, sharing contact and health information, and participating in evaluations. Patients were contacted and enrolled in surveys within 2-3 weeks of their telehomecare start date. Health care providers were included if they had referred a patient who had previously been enrolled in the telehomecare and/or had participated in delivering telehomecare as a provider or care administrator. For surveys, nurses needed at least 2 months of experience in providing care using telehomecare.

Patients aged 18 years or younger, those without an established diagnosis of COPD or HF, those unable or unwilling to provide verbal informed consent, and those demonstrating nonadherence to the telehomecare program were not eligible. The telehomecare nurse worked with each patient on a case-by-case basis to assess their willingness to participate in their own care and to determine if missed consultations and their reasons reflected overall nonadherence. Additionally, individuals who could not or would not use telehomecare equipment, or who did not have a regular caregiver to assist with the equipment if needed, were also excluded. Health care providers who did not practice in any of the 8 LHINs were excluded.

### Telehomecare Program Intervention

The telehomecare program intervention has been described extensively elsewhere [22]. Telehomecare aims to improve self-management skills for patients with HF and those with COPD by providing remote monitoring and nurse-delivered individualized health coaching sessions using the 5A’s (assess, advise, agree, assist, and arrange) Behavior Change Model [23,28,29]. Patients are provided with a touch-screen tablet, weight scale, blood pressure monitor, and pulse oximeter for weekday submission (Monday-Friday) of clinical information and self-report [23,30]. Telehomecare nurses monitor incoming patient information and respond to any deteriorating conditions or alerts triggered due to data received outside “normal” thresholds [31]. Following an alert, a nurse follows up and determines a course of action. If an alert impacts a patient’s well-being, telehomecare nurses immediately communicate with the patient’s care team.

### Outcome Measures and Data Collection

The outcomes for the longitudinal surveys were patient-perceived HRQoL, disease-management skills, perception of telehomecare, satisfaction with telehomecare, and caregiver-perceived strain. Cross-sectional surveys were conducted to assess nurse perception and satisfaction with telehomecare. Patients and caregivers were administered telephone surveys at baseline, 3, 6, and 12 months after starting the program from July 2016 to December 2019. Participants were contacted for a follow-up at least 4 (maximum 10) times and within 1 week before and 1-2 weeks after the scheduled date. Validated questionnaires were used to evaluate patients’ general and disease-specific HRQoL, satisfaction with telehomecare, self-management skills, and telehomecare perception. A complete list of survey instruments and details can be found in Multimedia Appendix 1.

### Questionnaires

#### Minnesota Living With Heart Failure Questionnaire

The Minnesota Living with Heart Failure (MLHF) is a 21-item questionnaire that evaluates the impact of frequent physical and emotional symptoms in patients with HF, with a higher score representing a worse impact on HRQoL [32]. The minimal clinically important difference (MCID) has been estimated to be 2.56 points in the physical domain, 0.98 points in the emotional domain, and 4.84 points in the overall domain [33].

#### Seattle Obstructive Lung Disease Questionnaire

The Seattle Obstructive Lung Disease Questionnaire (SOLD) is designed to monitor HRQoL in patients with COPD using 29 items and 4 scales. Larger scores in each dimension reflect a greater perception of HRQoL [34]. A change of approximately 5 points was observed for the MCID [34].

#### EQ-5D

The EQ-5D is a generic HRQoL measure which consist of an index score (0-1) and a visual analog scale (VAS; 0-100), with scores ranging from worst (0) to best health (1 or 100, respectively) [35].

#### Short-Form-12

The Short-Form-12 (SF-12) version 1 measures general HRQoL using 2 composite domains, with scores ranging from 0, the lowest, to 100, which represents the highest level of health in the physical component score (PCS) and mental component score (MCS) summary dimensions [36]. Within patients with HF, an MCID of 1.26 points in the PCS and 2.28 points in the MCS was reported [33]. There is limited information on the MCID for patients with COPD using the SF-12 version 1.

#### Chronic Disease Self Efficacy Scales Questionnaire

The Chronic Disease Self Efficacy Scales (SES) Questionnaire measures disease management skills with 6 items on a scale of 1-10, with higher scores indicating an improved level of self-confidence [37].

#### Telemedicine Perception Questionnaire

The Telemedicine Perception Questionnaire (TMPQ) measures patient perception of telehomecare and includes 17 items, with higher scores indicating a more positive perception of telehomecare [38].

#### Client Satisfaction Questionnaire

The Client Satisfaction Questionnaire–4 (CSQ-4) measures client satisfaction with program services, with higher scores indicating greater satisfaction [39].

#### Modified Caregiver Strain Index

Caregivers’ perceived strain was captured using the Modified Caregiver Strain Index (MCSI) with higher scores ranging from 0 (no strain) to 26 (highest level of strain) [40].

#### Nurse Satisfaction Questionnaire

Cross-sectional surveys using the Nurse Satisfaction Questionnaire (NSQ) were conducted with the nurses involved in the program to capture their satisfaction levels with telehomecare and perceived quality of care. The NSQ was administrated from August 2016 to March 2018 and scored on a 1-10 (best) scale.

### Data Missingness

Missing values at the survey level were imputed for the MLHF using its predetermined methods to neutralize the effects of missing values when calculating and comparing changes in scores [41]. Item number 10 within the MLHF was found to have a large proportion of item-level missingness, likely due to the item reflecting sexual activities. The TMPQ can only be scored when all items have been completed. Responses missing completely at random for the TMPQ were imputed using the last recorded observation for that question carried forward [42]. Missing values within the SOLD, SES, CSQ-4, MCSI, and NSQ were not imputed within the dataset, as this did not impact the ability to score the surveys. Missing values in the SF-12 were not imputed, and incomplete responses were not analyzed.

### Statistical Analysis

All analyses were performed using SAS Statistical Software (version 9.4, SAS Institute Inc). Descriptive statistics were used to summarize patient, provider, and caregiver survey data. Continuous variables were described using means and SDs. Categorical variables were described using counts and percentages. The entire cohort of patients was used to analyze the survey data for the SF-12, EQ-5D, SES, TMPQ, and CSQ-4. To look at telehomecare’s impact on the varying disease types (HF vs COPD), analyses using the MLHF and SOLD were performed using the respective patient cohorts. Repeated measures of the patient and caregiver surveys for continuous values were analyzed using generalized linear mixed models to determine if outcome measures of HRQoL, client satisfaction, telemedicine perception, and self-management scores were changing over time. A separate analysis was conducted for each outcome measure. The models were selected using the Akaike Information Criterion and used Restricted Maximum Likelihood estimation methods. Covariates included LHIN, disease type, age, gender, time, and the interactions between these variables of interest (time × diagnosis and time × gender). The Kenward-Roger approximation was used for the degrees of freedom, and the α level was set at 0.05 for all parameter tests [43]. EQ-5D-3L utility index scores were calculated using the Canadian value set [44]. Additionally, to address potential response bias, patient demographics of participants who consented and those who declined participation were analyzed using chi-square and 2-tailed Welch *t* tests.

## Results

### Study Enrollment

**Figure 1:**
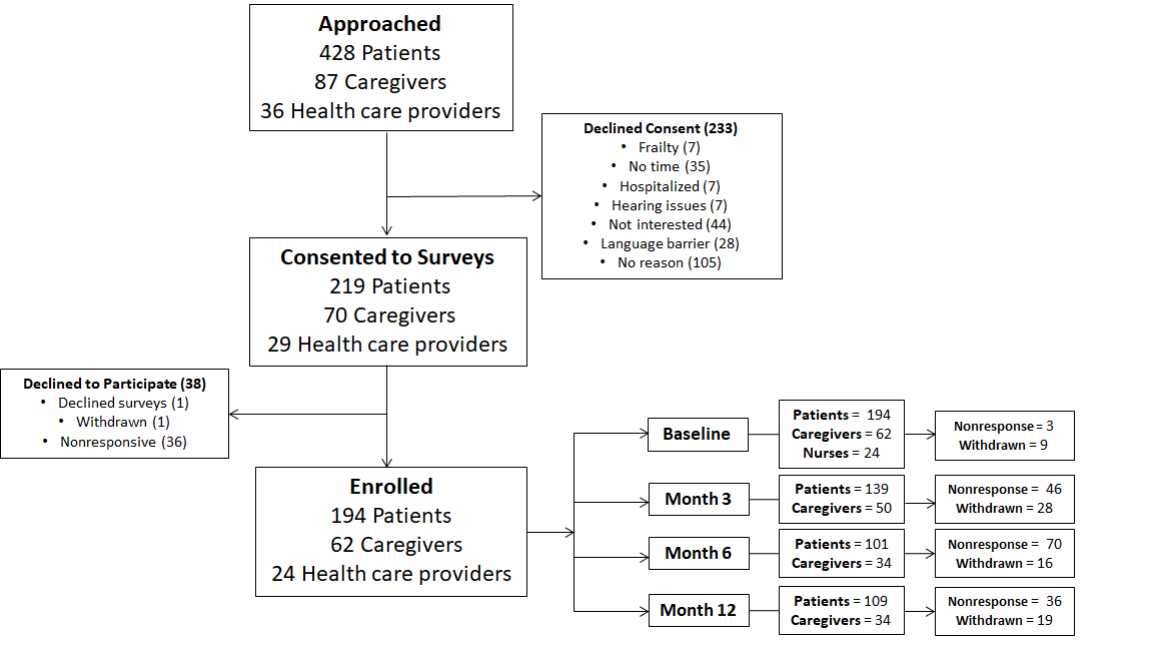
Patient flowchart.

Between July 2016 and December 2019, a total of 318 participants consented to take part in the study. Of those, 38 participants were excluded because they subsequently declined the surveys, withdrew, and were nonresponsive when contacted. In total, 194 patients (HF, n=117; COPD, n=77), 62 caregivers, and 24 nurses were enrolled in the program. An overall participant response rate of 51% (280/551) was observed. Figure 1 outlines the flow of participants through the study. The average age of patients with HF and those with COPD who participated in the surveys was 71 (SD 11.3) years and 70 (SD 11.1) years, respectively, and 52% (100/194) were men.

To assess potential response bias, we examined the characteristics of patients who consented to surveys and those who declined. The declined group consisted of patients who refused participation, whether they were approached directly or identified through the Patient Management and Monitoring System. There were no differences seen in relation to gender and condition; however, patients who declined to participate in surveys were older adults (Table 1). There is a possibility that older patients may have been in worse health and may not have benefited from being enrolled in telehomecare.

**Table 1:** Demographic characteristics of patients who consented and declined telehomecare participation.

Category and demographic			Consented (n=218)		Declined (n=402)		*P* value	
**Condition, n (%)**								.85
	HF^a^	129 (59.2)		241 (60)				
	COPD^b^	89 (40.8)		161 (40)				
**Gender, n (%)**								.76
	Male	108 (49.5)		194 (48.3)				
	Female	110 (50.5)		208 (51.7)				
**Age (years)**								<.001
	Missing, n	4		3				
	Mean (SD)	69.6 (11.4)		74.6 (11.2)				
	Range	29.0-98.0		30.0-102.0				

^a^HF: heart failure.

^b^COPD: chronic obstructive pulmonary disease.

### Survey Administration

We administered longitudinal survey instruments at baseline, month 3, month 6, and month 12 for patients and caregivers; however, not all participants completed the surveys at all timepoints. Cross-sectional surveys were administered to nurses with at least 2 months of experience in providing care using telehomecare. As noted in Table 2, the administration of surveys declined over the study duration due to participants being nonresponsive or withdrawing at various timepoints; a patient could complete the baseline surveys but fail to complete subsequent follow-ups. Across each instrument 40% (47/117; MLHF) to 42% (47/112; SES, TMPQ, and CSQ) of patients with HF completed surveys at all timepoints, 34% (26/77; SOLD) to 36% (27/76; SES, TMPQ, and CSQ) of patients with COPD completed surveys at all timepoints, and 37% (23/62; MCSI) of informal caregivers completed surveys at all timepoints.

**Table 2:** Complete survey administrations by population.

Population and instrument			Baseline, n		Month 3, n		Month 6, n		Month 12, n		Completed all timepoints, %
**Patients with heart failure**											
	MLHF^a^	117		87		60		68		40	
	EQ-5D	117		87		60		68		40	
	SF-12^b^	116		86		60		63		41	
	SES^c^	112		85		60		68		42	
	TMPQ^d^	113		86		59		66		42	
	CSQ^e^	113		85		59		64		42	
**Patients with chronic obstructive pulmonary disease**											
	SOLD^f^	77		51		40		41		34	
	EQ-5D	77		52		41		41		34	
	SF-12	77		51		40		39		35	
	SES	76		50		40		41		36	
	TMPQ	76		52		40		41		36	
	CSQ	76		52		40		41		36	
**Informal caregivers**											
	MCSI^g^	62		50		34		34		37	
**Nurses**											
	NSQ^h^	24		N/A^i^		N/A		N/A		N/A	

^a^MLHF: Minnesota Living with Heart Failure.

^b^SF-12: Short-Form-12.

^c^SES: Self Efficacy Scales.

^d^TMPQ: Telemedicine Perception Questionnaire.

^e^CSQ: Client Satisfaction Questionnaire.

^f^SOLD: Seattle Obstructive Lung Disease Questionnaire.

^g^MCSI: Modified Caregiver Strain Index.

^h^NSQ: Nurse Satisfaction Questionnaire.

^i^N/A: not applicable.

### Findings

#### MLHF

A total of 117 patients with HF completed the MLHF, contributing 332 observations across all timepoints. From baseline to month 12 the overall domain scores ranged from 44.6 (SD 23.8) to 21.5 (SD 15.7), physical domain scores from 20.3 (SD 10.8) to 12.2 (SD 8.2), and emotional domain scores from 10.4 (SD 7.4) to 3 (SD 3.3), indicating an improved (lower) score over time in each domain (Table 3). When adjusting for age and gender within the overall, physical, and emotional domains patients, with HF were shown to significantly improve over the 12-month follow-up (−18.37, *P*<.001; −5.77, *P*<.001; and −6.63, *P*<.001), respectively. Within the overall and physical domains, women were shown to have higher scores (worse health) than men (10.8, *P*=.01; 4.4, *P*=.02, respectively). However, within the overall and physical domains, women were shown to have significantly lower scores than men at month 12 (−9.53, *P*=.03; −5.42, *P*=.01, respectively), indicating a greater improvement in health over the study period. Across each timepoint and domain, the MCID was observed, indicating a clinical improvement in HRQoL for patients with HF receiving telehomecare (Table 4).

**Table 3:** Mean scores of survey instruments.

Instrument, domain, and diagnosis				Baseline, mean (SD)		Month 3, mean (SD)		Month 6, mean (SD)		Month 12, mean (SD)
**MLHF^a^**										
	**Overall**									
		HF^b^	44.6 (23.8)		31.3 (24.7)		29.1 (21.4)		21.5 (15.7)	
	**Physical**									
		HF	20.3 (10.8)		15.5 (11.2)		14.8 (10.1)		12.2 (8.2)	
	**Emotional**									
		HF	10.4 (7.4)		5.4 (5.7)		4.9 (4.7)		3 (3.3)	
**SOLD^c^**										
	**Physical function**									
		COPD^d^	36.6 (20)		39.1 (22)		39.6 (22.6)		42.5 (21.3)	
	**Emotional function**									
		COPD	65.2 (22.8)		70.4 (20.8)		69.4 (20.9)		67.2 (19.4)	
	**Coping skills**									
		COPD	66.8 (21.7)		72.2 (20.1)		73.5 (23.8)		72.6 (18.4)	
	**Treatment satisfaction**									
		COPD	66.7 (18.8)		73.9 (13.8)		74.5 (16.2)		68.8 (18.4)	
**EQ-5D**										
	**Index**									
		HF	0.69 (0.18)		0.71 (0.22)		0.69 (0.19)		0.69 (0.19)	
		COPD	0.70 (0.19)		0.68 (0.24)		0.70 (0.16)		0.68 (0.16)	
	**VAS^e^**									
		HF	55.6 (19.9)		56.9 (18.6)		54.7 (17.7)		55.4 (15.6)	
		COPD	65 (19)		59.4 (18.1)		57.4 (17.8)		55.1 (17.9)	
**SF-12^f^**										
	**PCS^g^**									
		HF	32.4 (9.8)		36.2 (11.5)		34.4 (10.4)		33.9 (11.3)	
		COPD	33.8 (9.2)		34.5 (11.4)		33.3 (10.6)		35.6 (9.9)	
	**MCS^h^**									
		HF	47.8 (9.2)		47.9 (9.6)		49.5 (7.6)		50.0 (6.5)	
		COPD	49.3 (10.4)		48.3 (9.7)		48.3 (9.5)		47.6 (9.3)	
**SES^i^**										
		HF	6.2 (2.0)		6.3 (1.8)		6.3 (1.8)		6.0 (1.8)	
		COPD	6.7 (2.1)		6.2 (1.7)		6.2 (1.7)		6.1 (1.9)	
**TMPQ^j^**										
		HF	62.1 (5.2)		64.6 (7.2)		64.7 (7.0)		61.7 (5.4)	
		COPD	62.8 (4.6)		65.5 (6.3)		65.9 (6.4)		62.9 (3.6)	
**CSQ^k^**										
		HF	13.6 (2.1)		13.9 (2.2)		14 (2.1)		13.3 (2.1)	
		COPD	14.1 (1.7)		14.5 (1.5)		14.5 (1.6)		13.8 (1.8)	
**MCSI^l^**										
	Caregiver strain			10.7 (6.2)		11.3 (6.4)		11.5 (6.3)		10.3 (5.9)
**NSQ^m^**										
	Nurse satisfaction			6.7 (1.5)		N/A^n^		N/A		N/A
	Quality of care			6.1 (0.5)		N/A		N/A		N/A

^a^MLHF: Minnesota Living with Heart Failure.

^b^HF: heart failure.

^c^SOLD: Seattle Obstructive Lung Disease Questionnaire.

^d^COPD: chronic obstructive pulmonary disease.

^e^VAS: visual analog scale.

^f^SF-12: Short-Form-12.

^g^PCS: physical component score.

^h^MCS: mental component score.

^i^SES: Self Efficacy Scales.

^j^TMPQ: Telemedicine Perception Questionnaire.

^k^CSQ: Client Satisfaction Questionnaire.

^l^MCSI: Modified Caregiver Strain Index.

^m^NSQ: Nurse Satisfaction Questionnaire.

^n^N/A: not applicable.

**Table 4:** Estimated change over time for survey domains.

Instrument, domain, and variable				Baseline		Month 3		Month 6		Month 12
				Estimate (*P* value)		Estimate (*P* value)		Estimate (*P* value)		Estimate (*P* value)
**MLHF^a^**										
	**Overall (n=115)**									
		Age	–0.08 (.58)		N/A^b^		N/A		N/A	
		Time	N/A		–8.87 (.005)		–11.68 (<.001)		–18.37 (<.001)	
		Gender	10.80 (.01)		N/A		N/A		N/A	
		Time × gender	N/A		N/A		N/A		–9.53 (.03)	
	**Physical (n=115)**									
		Age	0.02 (.73)		N/A		N/A		N/A	
		Time	N/A		–3.35 (.03)		–4.54 (.004)		–5.77 (<.001)	
		Gender	4.40 (0.02)		N/A		N/A		N/A	
		Time × gender	N/A		N/A		N/A		–5.42 (.01)	
	**Emotional (n=115)**									
		Age	–0.03 (.48)		N/A		N/A		N/A	
		Gender	2.03 (.07)		N/A		N/A		N/A	
		Time	N/A		–3.91 (<.001)		–4.93 (<.001)		–6.63 (<.001)	
**SOLD^c^**										
	**Treatment satisfaction (n=76)**									
		Age	–0.02 (.94)		N/A		N/A		N/A	
		Gender	6.80 (.10)		N/A		N/A		N/A	
		Time	N/A		9.50 (.01)		11.22 (.004)		N/A	
	**Physical (n=76)**									
		Time	N/A		–0.85 (.83)		–0.01 (.99)		6.11 (.14)	
		Age	–0.16 (.56)		N/A		N/A		N/A	
		Gender	–6.81 (.16)		N/A		N/A		N/A	
	**Coping (n=76)**									
		Time	N/A		0.32 (.94)		1.33 (.78)		5.88 (.23)	
		Age	0.20 (.45)		N/A		N/A		N/A	
		Gender	–1.79 (.72)		N/A		N/A		N/A	
**EQ-5D**										
	**VAS^d^ (n=190)**									
		Age	–0.14 (.17)		N/A		N/A		N/A	
		Gender	–1.59 (.48)		N/A		N/A		N/A	
		Diagnosis	9.47 (.001)		N/A		N/A		N/A	
		Time × diagnosis	N/A		–7.20 (.04)		–6.54 (.08)		–9.78 (.005)	
**SF-12^e^**										
	**PCS^f^ (n=190)**									
		Age	–0.06 (.33)		N/A		N/A		N/A	
		Gender	–1.67 (.22)		N/A		N/A		N/A	
		Time	N/A		3.86 (<.001)		2.69 (.02)		2.01 (.06)	
		Time × diagnosis	N/A		–3.24 (.05)		–2.24 (.21)		0.08 (.96)	
	**MCS^g^ (n=190)**									
		Age	0.03 (.68)		N/A		N/A		N/A	
		Time	N/A		N/A		N/A		2.25 (.05)	
		Gender	–13.89 (.05)		N/A		N/A		N/A	
		Time × diagnosis	N/A		–1.06 (.55)		–2.68 (.16)		–3.53 (.05)	
**TMPQ^h^**										
	**Telehomecare perception (n=186)**									
		Time	N/A		2.50 (<.001)		2.80 (<.001)		N/A	
		Age	–0.10 (.002)		N/A		N/A		N/A	
		Gender	0.08 (.91)		N/A		N/A		N/A	
		Time × diagnosis	N/A		–0.12 (.92)		0.42 (.72)		–0.29 (.80)	
**CSQ^i^**										
	**Client satisfaction (n=185)**									
		Time	N/A		N/A		N/A		–0.44 (.05)	
		Age	–0.04 (.002)		N/A		N/A		N/A	
		Gender	–0.14 (.59)		N/A		N/A		N/A	
		Time × diagnosis	N/A		–0.03 (.92)		–0.04 (.90)		0.15 (.67)	

^a^MLHF: Minnesota Living with Heart Failure.

^b^N/A: not applicable.

^c^SOLD: Seattle Obstructive Lung Disease Questionnaire.

^d^VAS: visual analog scale.

^e^SF-12: Short-Form-12.

^f^PCS: physical component score.

^g^MCS: mental component score.

^h^TMPQ: Telemedicine Perception Questionnaire.

^i^CSQ: Client Satisfaction Questionnaire.

#### SOLD

A total of 77 patients with COPD completed the SOLD, contributing 209 observations across all timepoints. From baseline to month 12 SOLD physical function scores varied between 36.6 (SD 20) to 42.5 (SD 21.3), emotional function scores from 65.2 (SD 22.8) to 67.2 (SD 19.4), coping skills scores from 66.8 (SD 21.7) to 72.6 (SD 18.4), and treatment satisfaction scores from 66.7 (SD 18.8) to 68.8 (SD 18.4), indicating an improved (higher) score over time in each domain (Table 3). Considering all covariates (time, age, and gender) within each SOLD domain, only treatment satisfaction at months 3 and 6 was statistically significant (*P*=.01; *P*=.004, respectively), indicating higher satisfaction with treatments while enrolled in telehomecare. MCIDs were observed in the physical function and coping skills dimensions over the 12-month follow-up, as well as at months 3 and 6 of the treatment satisfaction dimensions (Table 4).

#### EQ-5D

HF index scores remained consistent from baseline to month 12, whereas COPD index scores declined over the same period. Index scores from baseline to month 12 ranged from 0.69 (SD 0.18) to 0.69 (SD 0.19) for patients with HF and 0.70 (SD 0.19) to 0.68 (SD 0.16) for patients with COPD (Table 3). From baseline to month 12 the mean unadjusted VAS scores ranged from 55.6 (SD 19.9) to 55.4 (SD 15.6) for patients with HF and 65 (SD 19) to 55.1 (SD 17.9) for patients with COPD. HF VAS scores remained consistent from baseline to month 12, with COPD VAS scores declining during that time. In relation to the VAS, patients with COPD had significantly higher scores than patients with HF (*P*=.001). After adjusting for time, age, and gender patients with COPD had significantly lower EQ-5D VAS scores in comparison to patients with HF at month 3 and month 12 (–7.20, *P*=.03; –9.78, *P*=.005), respectively. There were no significant differences between the index scores of patients with COPD and those with HF when adjusting for time, gender, and age (Table 4).

#### SF-12

A total of 193 patients responded completely to the SF-12 questionnaires, contributing a total of 532 administrations at all timepoints. From baseline to month 12, the mean unadjusted PCS scores ranged from 32.4 (SD 9.8) to 33.9 (SD 11.3) for patients with HF and 33.8 (SD 9.2) to 35.6 (SD 9.9) for patients with COPD, indicating an improved (higher) score over time for both conditions. MCS scores varied from 47.8 (SD 9.2) to 50.0 (SD 6.5) for patients with HF and 49.3 (SD 10.4) to 47.6 (SD 9.3) for patients with COPD. HF MCS scores improved from baseline to month 12; however, COPD MCS scores declined during that time (Table 3). In relation to the PCS, there was an improvement in scores at month 3 and month 6 (*P*<.001; *P*=.02). However, at month 3, patients with COPD were shown to have significantly lower scores (worse health) than patients with HF (–3.24, *P*=.05). Within the MCS there was an improvement in scores at month 12 (*P*=.05), and patients with COPD at month 12 were shown to have significantly lower scores (worse health) than patients with HF (–3.53, *P*=.05). Women were also shown to have significantly lower MCS scores than men, regardless of condition (–13.89, *P*=.05). MCIDs were achieved in the HF PCS scores across each timepoint, indicating a clinical improvement in the health of patients with HF (Table 4).

#### Chronic Disease SES Questionnaire

Patient self-management scores were consistently high throughout the program and did not improve over time after adjusting for age, gender, and diagnosis. Additionally, patients with HF and those with COPD did not express any significant differences in their ability to self-manage disease symptoms.

#### TMPQ

Throughout all timepoints, patients reported high levels of satisfaction with telehomecare as reported on the TMPQ summary scores (Table 3). The perception of telehomecare improved at month 3 and month 6; however, it is important to note that patients were no longer on the program at month 12. The age of patients may have a role to play in their perception of telehomecare. We observed that as patients’ ages increased in years, scores of the TMPQ were significantly lower (–0.10, *P*=.002; Table 4).

#### CSQ

CSQ scores were shown to significantly decline at month 12, likely due to patients not being on the program since month 6. The age of patients may have a role to play in their satisfaction with telehomecare services. Our results indicated that as patients’ age increased in years, scores on the TMPQ and CSQ were significantly lower (–0.04, *P*=.002; Table 4).

#### MCSI

A total of 62 caregivers provided 180 observations across all timepoints for the MCSI, and unadjusted scores from baseline to month 12 were 10.7 (SD 6.2) to 10.3 (SD 5.9), indicating a reduction in caregiver strain. Overall, caregivers had relatively low strain scores across all timepoints and did not improve over time after adjusting for age, gender, and diagnosis.

#### NSQ

Nurses reported moderate levels of satisfaction and perceived quality of care with telehomecare across all LHINs, with an overall mean of 6.7 (SD 1.5) and 6.1 (SD 0.5), respectively. A complete breakdown of the NSQ results can be found in the Multimedia Appendix 1.

## Discussion

### Principal Results

In this study, participation in telehomecare was shown to significantly improve perceived HRQoL of patients with HF across all timepoints using the MLHF. An MCID was observed in each domain of the MLHF, inferring a clinical improvement in HRQoL of patients with HF. Additionally, MCIDs were observed in the SOLD, indicating that patients with COPD experienced clinical improvements in physical, coping, and treatment satisfaction function. Results from the EQ-5D indicated that patients with COPD had significantly higher VAS scores than patients with HF. Furthermore, MCIDs were detected in the SF-12 HF PCS scores, indicating a clinical improvement in the health of patients with HF. Using the SES instrument, patients reported being confident in self-managing their diseases throughout the entire study duration, and satisfaction with telehomecare was consistently positive using both the TMPQ and CSQ. Patients who were involved in telehomecare perceived it to be important, but once they completed the program and upon follow-up, their perception of telehomecare was understandably lower. While satisfaction and perception of telehomecare were highly rated at months 3 and 6, as time went on, lower scores were reported by older patients. This could be due to the older patients in the program being in poorer health and having a more difficult time self-managing their comorbid conditions.

### Comparison With Prior Work

There are only a few studies which provide patient-reported outcomes as a primary measure or as well-explored secondary outcomes within the literature. The literature surrounding the use of telehealth interventions and their ability to impact patients’ HRQoL is inconsistent in definitively proving its value in patients with HF and those with COPD. A review examining telemonitoring in patients with HF and those with COPD further identified conflicting results on telehomecare’s impact on HRQoL [14].

### Telemedicine’s Impact on Patients With HF

Conclusions from the Telemedicine for Heart study identified in the review indicated that patients with HF who received telemonitoring improved their quality of life as measured by the Short-Form-36 (SF-36) [14,45]. Conversely, upon further review of the study, both groups’ scores were not significantly different from one another, SF-36 data were not reported completely, and some telemedicine group scores were worse than controls. Therefore, the conclusion that HRQoL was improved in patients with HF could not be supported in the Telemedicine for Heart study [45]. The Telemedicine for Heart study population (mean age 65.2, SD 9.9 years; 233/281, 83% men) varied from this telehomecare study as participants were, on average, younger and their study population was less diverse with only 48 (17%) women [45]. The BEAT-HF 180-day MLHF score (28.50), closely reflects the month 6 score observed in this telehomecare study (29.1, SD 21.4). Additionally, the BEAT-HF telehomecare intervention and patient population were similar to this study with a median age of 73 (range 62-84) years and 46.6% (664/1437) female participants. However, the BEAT-HF trial did not provide HRQoL data in full (no baseline data) or report any measures of dispersion, limiting the generalizability of this study’s findings [46]. The Medly telemonitoring intervention reported improvements in MLHF scores of patients with HF in comparison to controls over 6 months. The telemonitoring group was predominately men (78%) and was significantly younger (mean age 58, SD 15.5 years) than the telehomecare patient group. Additionally, based on the baseline MLHF overall domain scores of the telemonitoring group (53.2, SD 26.3), these patients had worse symptoms than those enrolled in telehomecare (44.6, SD 23.8) [47].

A Cochrane review of 11 telemonitoring studies that measured HRQoL in patients with HF stated that only 5 studies reported statistically significant improvements in HRQoL outcomes [48]. Among the studies that reported improvements in HRQoL of patients with HF, there are a few caveats to take into consideration. Antonicelli et al [49] reported improvements only in the SF-36 health perception subscale, but component scores remained nonsignificant. The Medicare Coordinated Care Demonstration (MCCD) Project improved SF-36 PCS, MCS, and MLHF overall, physical, and emotional domains of patients with HF. However, these improvements were not significantly different from the results seen in the control group [50]. MCCD patients received a similar remote monitoring program as those in telehomecare. However, their patients with HF were older (mean age 73, SD 8 years) than those enrolled in telehomecare and were predominately male (71/101, 70%). The MCCD MLHF domain scores at baseline, month 6, and after 1 year were also compared to MLHF scores observed in this study [50]. The TIM-HF study reported improvements in SF-36 physical functioning scores in the intervention group versus the control group at 12- and 24-month follow-up after receiving remote patient monitoring. The TIM-HF study patient population was younger than telehomecare, with a mean age of 66.9 (SD 10.8) years, and was less diverse, with primarily men (81%) enrolled [51]. Subsequently, the TIM-HF II study, with a mean age of 70 (SD 11) years, like telehomecare, reported no statistical differences in MLHF scores between remote patient monitoring and usual care from baseline to 12 months [52].

### Impact of Telemedicine on Patients With COPD

Two Cochrane reviews examining patients with COPD with a history of admissions for exacerbations concluded that telemonitoring provided inconclusive evidence of a benefit and did not improve patients’ quality of life compared to usual care [53,54]. The review of tele–health care interventions for COPD by McLean et al [55] identified only 2 studies which reported HRQoL using validated instruments. The meta-analysis in this review reported an imprecise pooled estimate, which indicated insufficient evidence to demonstrate a clear benefit of tele–health care interventions for patients with COPD. The included studies contained patients aged 69.4 (SD 6.5) years and those aged 70 (SD 9) years, which closely related to the telehomecare population [56,57]. The TeleCare North study was a large-scale trial aimed at establishing evidence of HRQoL in patients with COPD receiving tele–health care in Denmark and closely resembling telehomecare. TeleCare North reported nonsignificant differences between intervention and control groups when looking at SF-12 PCS and MCS scores over 12 months. Interestingly, the findings of the TeleCare North study suggest that tele–health care interventions may provide a psychological benefit and work to slow the deterioration of mental health in patients with COPD [58]. In comparing the unadjusted MCS scores from baseline to month 12 for patients with COPD, a similar reduction in change scores was observed (telehomecare, –1.7 vs TeleCare North, –1.5).

### HRQoL and Remote Patient Monitoring

These results reinforce the inconsistencies in the literature regarding telemonitoring improving HRQoL among patients with HF and those with COPD [58,59]. Using a disease-specific measure such as the MLHF may be more appropriate than the SF-12 or SF-36 in capturing significant changes in the health of patients with HF over time, while a more responsive disease-specific measure may need to be identified for longitudinal use in patients with COPD. Additionally, a greater emphasis may need to be placed on the MCID of HRQoL for patients with HF and those with COPD receiving telehomecare. Telehomecare seems to be well received by the majority of patients, but home telemonitoring may have more significant benefits to the HRQoL of patients with HF and those with COPD in mild to moderate disease states than those whose health has already begun to deteriorate [60]. These results provide more credence to the use of a risk stratification framework to identify patients with mild to moderate risk of hospitalization who should be directed to telehomecare [61,62]. Future research should focus on using disease-specific measures to assess HRQoL in patients and assessing change using validated MCIDs to identify meaningful change.

### Clinician and Caregiver Involvement in Telemedicine

For telehomecare to be readily accepted and implemented, health care providers must be on board and believe it brings tangible benefits to their patients. Engagement and collaboration from clinicians may be critical success factors for telehomecare, as clinicians are at the forefront of the care provided to patients, and they should be a part of designing the program. Additionally, telehealth interventions should be integrated into the clinician’s workflow, creating an environment that is conducive to the use of the equipment [62-64]. El-Dassouki et al [65] report how a telemedicine intervention can reduce uncertainty, reduce caregiver strain by easing their workload, and mitigate clinical knowledge requirements.

### Strengths and Limitations

A vital strength of the longitudinal surveys was the use of validated generic and disease-specific measures, which provided us with the opportunity to compare important changes in patient’s health over time. Using the MLHF and SOLD questionnaires allowed us to measure fundamental changes that patients deemed as important to their health status regarding their condition. The length of follow-up (12 months) was also an asset for the study because we were able to evaluate patients’ self-management skills and perception of telehomecare after being off the program for a considerable amount of time. This study has limitations, which warrant discussion. First, this study was a part of a comprehensive program evaluation, which did not have a control group to evaluate the impact of telehomecare compared to usual care. Second, we were unable to gain access to a proportion of the patient’s demographic information due to the migration of OTN telehomecare databases during this study’ period. Third, there was no longitudinal follow-up for the NSQ; scores were cross-sectional. Fourth, we were not notified each time a patient passed away within the program; consequently, not every patient death was explicitly documented. When a participant was found to have died, their status was recorded as a withdrawal. Lastly, we were unable to recruit a reasonable number of physicians to take part in the study and gain their perceptions of telehomecare, which is critically important in understanding its adoption by health care providers.

### Conclusion

Telehomecare was created to improve access to quality care and reduce health care use by delivering health care at a distance and providing tailored feedback. The OTN telehomecare program has demonstrated an ability to improve the HRQoL of patients with HF and those with COPD. However, the long-term sustainability of HRQoL improvements requires further investigation. Additionally, telehomecare has been shown to decrease informal caregiver perceived strain, and telehomecare nurses described moderate levels of satisfaction and perceived quality of care with telehomecare. Telehomecare seems to be well received by most patients with HF and those with COPD, but more research is needed into home telemonitoring to determine its value in improving HRQoL.

## Appendix

Multimedia Appendix 1 Description of survey instruments and nurse satisfaction questionnaire.

## Abbreviations

COPD: chronic obstructive pulmonary disease
CSQ-4: Client Satisfaction Questionnaire–4
HF: heart failure
HRQoL: health-related quality of life
LHIN: local health integration network
MCCD: Medicare Coordinated Care Demonstration
MCID: minimal clinically important difference
MCS: mental component score
MCSI: Modified Caregiver Strain Index
MLHF: Minnesota Living with Heart Failure
NSQ: Nurse Satisfaction Questionnaire
OTN: Ontario Telemedicine Network
PCS: physical component score
SES: Self Efficacy Scales
SF-12: Short-Form-12
SF-36: Short-Form-36
SOLD: Seattle Obstructive Lung Disease Questionnaire
STROBE: Strengthening the Reporting of Observational Studies in Epidemiology
TMPQ: Telemedicine Perception Questionnaire
VAS: visual analog scale
