# Supplementary material for: The Effect of Telehomecare on Patients’ Health-Related Quality of Life, Satisfaction, Disease Self-Management Skills, Provider Satisfaction, and Informal Caregiver Strain: Longitudinal Cohort and Cross-Sectional Study
Source: JMIR Form Res. 2026 Jan 15;10:e70809. doi: 10.2196/70809 (PMC12856394; doi:10.2196/70809)
Supplement: Multimedia Appendix 1 [file formative_v10i1e70809_app1.docx]

Supplemental Appendix

Contents

[Description of Survey Instruments 2](#_Toc208858658)

[Nurse Satisfaction Questionnaire 4](#_Toc208858659)

# Description of Survey Instruments

| Name of Instrument | Domains / Subscales (# of items) | Summary Scoring |
| --- | --- | --- |
| 12 - Item Short Form Survey (SF-12) | \| Physical Component Summary (6) Physical Functioning (2) Role Physical (2) Bodily Pain (1) General Health (1)  Mental Component Summary (6) Vitality (1) Social Functioning (1) Role Emotional (2) Mental Health (2) \| \| --- \| | The SF-12 survey is an abbreviated version of the SF-36 survey, which targets to measure health related to quality of life.  Twelve questions are combined, scored, and weighted to create 2 composite scales for physical and mental health status referred to as the Physical Component Summary (PCS) and the Mental Component Summary (MCS).  Composite scores range from 0 to 100, where a score of zero indicates the lowest level and 100 indicates the highest level of health. |
| EuroQol 5-Dimension (EQ-5D) | Mobility Self-care Usual Activities Pain/Discomfort Anxiety/Depression  Visual Analogue Scale  Each dimension has three levels: no problems, some problems, severe problems | EQ-5D health states can be converted into a single summary. The index can then be calculated by deducting the appropriate values/weights from 1, which is the value for full health.  The Visual analogue scale (VAS) records a patient’s self-rated health in relation to their own judgement of ‘Best imaginable health state’ and ‘Worst imaginable health state’. |
| Minnesota Living with Heart Failure Questionnaire (MLHF) | Overall Score (21)  Physical Descriptive Factor (8)  Emotional Descriptive Factor (5)  6-point Likert scale / from 0 ‘no impairment’ to 5 ‘very much impairment’ | The MLHF questionnaire assesses the impact of frequent physical symptoms of heart failure - shortness of breath, fatigue, swollen ankles and difficulty sleeping. Additionally, the MLHF evaluates the effects of heart failure on physical and social functions.  Simple summation of responses. Total score (min = 0, max = 105), physical dimension (min= 0, max = 40) emotional dimension (min = 0, max = 25); the higher the summed score, the worse is the impact of heart failure on a patient’s quality of life. |
| Seattle Obstructive Lung Disease Questionnaire (SOLD) | Physical Functioning Emotional Functioning Coping Skills Treatment Satisfaction  The higher the score on each dimension of the questionnaire, the better is the health related quality of life in patients. | The physical function scale assesses the degree of dyspnea and the extent of physical limitation. The emotional function scale measures the impact of the disease on patient’s psychological well-being. The coping skills scale measures self-efficacy which reflects a patient’s conviction regarding his/her ability to achieve a certain outcome and is an important determinant of successful behavior change.  The fourth dimension, treatment satisfaction, measures how satisfied patients are with the therapy they are getting for COPD.  The raw score for each scale is calculated by summing the responses to the items on that scale. Raw scores are then transformed to a normalized score (ranging from 0-100) by subtracting the lowest possible score from the raw score and then dividing the resulting score by the range of possible scores and multiplying by 100. |
| Telemedicine Perception Questionnaire (TMPQ) | The TMPQ measures patient perceptions of Telehomecare and includes 17 items using a 5-point Likert scale that covers several degrees of agreement (from 1 for ‘strongly disagree’ to 5 for ‘strongly agree’). Items worded in a negative manner need to have a reversed scoring (1 for ‘strongly agree’, 5 for ‘strongly disagree’). | Items address the effect of Telehomecare on the quality of and access to health care, the issues of time and money (including time savings for the patient and/or the nurse, reduction of costs for the patient and/or the health care agencies), and factors related to the conduct of a virtual visit (including ease of equipment use, equal acceptability of “virtual” and real visit, protection of privacy and confidentiality, lack of physical contact, reduced sense of intimacy, and patient’s ability to explain medical problems in a virtual visit).  The individual scores can range from 17 to 85. The higher the total score the more positive is the overall perception a patient has of Telehomecare. |
| Chronic Disease Self-Efficacy Scale (SES) | Symptom control Role function  Emotional functioning Communicating with physicians. | The score for the scale is the mean of the six items. If more than two items are missing, do not score the scale. Each item has a scale of 0-10 with higher scores indicating a better level of self-confidence and disease management. |
| Client Satisfaction Questionnaire (CSQ-4) | 4-item questionnaire which elicits the client’s perspective on the value of services received. | CSQ- 4 is designed to measure client satisfaction with services (item scores produce a range of 4 to 16, scores are summed with higher scores indicating greater satisfaction). |
| Modified Caregiver Strain Index (MCSI) | Employment Financial Physical Social Time | The MCSI is a tool that can be used to quickly screen for caregiver strain with long-term family caregivers. It is a 13-question tool that measures strain related to care provision  Scoring is 2 points for each ‘yes’, and 1 point for each ‘sometimes’ response. The higher the score, the higher the level of caregiver strain. Scores can range from 0 (no strain) to 26 (Highest level of strain) |
| Nurse Satisfaction Questionnaire (NSQ) | Satisfaction with System (5)  Perceived Effects on Quality of Care (10)  Effects on Individual and Group Productivity (4) | Mean (SD) on a scale from 1-10. Higher scores reflect agreement and improved performance |

# Nurse Satisfaction Questionnaire

Table 1: Nurse Satisfaction with Telehomecare across all LHINs

| **Do you feel that the Telehomecare system in your unit:** | **Mean (SD) ON A  SCALE OF 1-10** |
| --- | --- |
| Is easy to use? | 6.7 (1.9) |
| Requires computing knowledge that you already have? | 8.5 (1.3) |
| Is clear and understandable? | 7.8 (1.5) |
| Rarely goes temporarily offline? | 5.2 (2.0) |
| Has a rapid response time? | 5.3 (1.7) |
| Overall mean | 6.7 (1.5) |
| 1 = Strongly Disagree and 10 = Strongly Agree. | |

Table 2: Perceived Effects of Telehomecare on Quality of Care Across all LHINs

| **To what extent does the Telehomecare system allow you to better perform the following tasks?** | **Mean (SD) ON A  SCALE OF 1-10** |
| --- | --- |
| Review a patient's clinical condition. | 6.3 (1.4) |
| Find specific information in the patient's file. | 6.0 (2.0) |
| Quickly grasp the information in the patient records. | 5.7 (1.8) |
| Follow changes in the patient's clinical condition over time. | 6.2 (1.8) |
| Avoid repeatedly entering information in the patient's file. | 5.3 (1.7) |
| Draft nurse observation notes. | 6.6 (1.4) |
| Update the nursing care plan. | 6.0 (1.7) |
| Ensure that all the clinical information is complete at the end of each day. | 7.2 (1.9) |
| Provide or obtain information on the patient's clinical condition during shift changes. | 5.5 (2.1) |
| Update therapeutic care plans. | 5.8 (1.8) |
| Overall mean | 6.1 (0.5) |
| 1 = Poor Performance and 10 = Excellent Performance. | |
